# Accuracy and Precision of Femoral and Tibial Bone Resections Using Manual Unrestricted Kinematic Alignment in Total Knee Arthroplasty: A Retrospective Digital Caliper Study

**DOI:** 10.3390/jcm15093506

**Published:** 2026-05-03

**Authors:** Charles Riviere, Loic Villet

**Affiliations:** 1 Bordeaux Arthroplasty Research Institute (BARI), 6 Rue Georges Negrevergne, 33700 Mérignac, France; loic.villet@bari-arthroplasty.com; 2 Clinique du Sport Bordeaux Mérignac, 4 Rue Georges Negrevergne, 33700 Mérignac, France

**Keywords:** total knee arthroplasty, kinematic alignment, unrestricted, caliper-verified, resection thickness, tibial resection, accuracy, precision

## Abstract

**Purpose:** To evaluate the accuracy and precision of both femoral and tibial bone resections in unrestricted kinematic alignment total knee arthroplasty (uKA TKA) performed with manual instrumentation, using postoperative digital caliper measurements. **Methods:** A retrospective study analyzing prospectively collected data on femoral and tibial resection thickness in 73 patients undergoing primary uKA TKA. Femoral cuts were performed with manual KA-optimized instrumentation in all cases. Tibial cuts were performed manually in 58 cases and with patient-specific instrumentation (PSI) in 15; PSI tibial resections were excluded from tibial analyses. Postoperatively, resection thickness was measured using a digital vernier caliper (0.2 mm resolution) at predefined sites: distal medial femur (DMF), distal lateral femur (DLF), posterior medial femur (PMF), posterior lateral femur (PLF), medial tibial plateau (MTP), and lateral tibial plateau (LTP). Resection error was defined as measured minus target thickness (mm). Accuracy was reported as mean signed error; precision as SD of signed error; absolute errors and error class distributions were also reported. Postoperative measurements reflect the accuracy and precision of the initial manual tibial resections, excluding any subsequent corrective cuts. **Results:** A total of 408 measurements were analyzed (292 femoral, 116 tibial). Mean signed error across resections was low and consistently negative (−0.15 to −0.31 mm), with infra-millimetric precision (SD 0.45 to 0.73 mm). Mean absolute errors remained low across sites (0.35 to 0.62 mm). The proportion of errors outside ±0.5 mm ranged from 21.1% (PLF) to 44.4% (LTP) and those outside ±1.0 mm from 1.4% (DMF) to 18.5% (LTP). No errors exceeded ±2.0 mm. **Conclusions:** Manual caliper-verified unrestricted KA TKA achieved high accuracy and precision for both femoral and tibial resections. However, these findings do not establish superiority over other techniques and do not account for final implant position, soft-tissue balance, or clinical outcomes. This study provides quantitative data on tibial resection accuracy in uKA TKA and may serve as a benchmark for evaluating the performance of technology-assisted techniques.

## 1. Introduction

Kinematic alignment (KA) is a surgical technique for total knee arthroplasty (TKA) that aims to minimize alteration to the native femorotibial joint line, soft-tissue balance, and knee kinematics [1]. Compared with conventional mechanical alignment (MA), which applies uniform alignment targets across patients, frequently altering the native joint line whilst requiring ligament releases to achieve balance [2,3], KA represents a paradigm shift toward patient-specific anatomy. Current evidence suggests that KA is biomechanically sound and clinically effective at mid-term follow-up, with improvements in joint awareness and patient satisfaction and with no sign of catastrophic failure [4]. Nevertheless, concerns persist regarding both the safety of restoring extreme native knee anatomy or laxity, in addition to the optimal instrumentation for executing the technique.

Unrestricted KA (uKA) TKA aims to reproduce the native femorotibial joint line and balance without predefined constraints on component alignment. This approach contrasts with restricted KA (rKA), in which component positioning is modestly adjusted in the presence of extreme anatomy (e.g., severe tibia vara or distal femoral valgus) [5]. It also differs from functional alignment (FA), where the final implant position represents a compromise dictated by the anatomy of the distal femur, proximal tibia, and trochlea, as well as soft-tissue laxity [6]. Although acceptable alignment boundaries and lateral compartment laxity limits are actively being investigated, there is no evidence to date which supports the superiority of one alignment philosophy over another. uKA, rKA, and FA TKA have each demonstrated promising clinical outcomes; however, direct comparisons remain unavailable due to the absence of comparative studies.

Whether uKA TKA is more accurately performed using manual techniques or assistive technologies, such as patient-specific instrumentation (PSI), computer navigation, or robotics, remains debated. Manual execution of uKA TKA, referred to as the caliper-verified KA technique, can be performed using dedicated KA-optimized instrumentation [7]. Several peer-reviewed studies have reported sub-millimetric accuracy of initial femoral resections using this technique; however, these investigations focused exclusively on femoral resections [8,9]. Despite the critical role of the tibia in joint line restoration, ligament balance, and load distribution, tibial resection thickness accuracy has not been quantitatively assessed in uKA TKA. Moreover, prior studies relied on intraoperative measurements using analog vernier calipers, which require visual scale interpretation and may introduce observer-dependent variability. Digital calipers provide higher measurement resolution and eliminate scale interpolation and rounding, but their intraoperative use is limited by sterilization constraints.

The purpose of this study was therefore to evaluate the accuracy and precision of both femoral and tibial resections in uKA TKA using postoperative digital caliper measurements. We hypothesized that digital caliper-verified uKA TKA would achieve sub-millimetric accuracy and high precision for both femoral and tibial resections.

## 2. Materials and Methods

### 2.1. Study Design and Population

This retrospective study analyzed prospectively collected data on femoral and tibial resection thickness in 73 patients undergoing primary uKA TKA for end-stage knee osteoarthritis between June and December 2025 (Table 1). All procedures were performed by two surgeons with extensive experience in KA TKA. Femoral cuts were performed using manual KA instrumentation (Medacta, Castel San Pietro, Switzerland) in all cases. Tibial resections were performed using manual instrumentation in 58 cases and PSI in 15 cases (hybrid technique). The choice between PSI and manual tibial cutting was determined by surgeon preference and logistical considerations and was not influenced by knee anatomy or case complexity. Because the study aimed to assess the performance of the manual caliper-verified technique, tibial resections performed with PSI were excluded from tibial analyses. Institutional review board approval was obtained prior to study initiation (CERC-VS-2026-03-2), and all patients provided informed consent.

### 2.2. Surgical Technique (Caliper-Verified uKA TKA)

Femoral resections were performed first using KA-optimized manual instrumentation with a measured-resection strategy based on distal and posterior referencing. The objective was true resurfacing, removing a composite thickness of bone and cartilage matching the implant thickness: 9 mm for distal femur, 8 mm for posterior femur, and 10 mm minimum tibial implant thickness in the present study (implant system: GMK-SpheriKA^®^, Medacta, Castel San Pietro, Switzerland).

For the distal femoral cut, sagittal orientation (flexion–extension) was set using a short intramedullary guide. Coronal orientation (varus–valgus) and cut height were defined by distal referencing, with the cutting jig positioned in direct contact with the most distal points of the femoral condyles, without screwing the jig onto the distal femur. When full-thickness cartilage wear was present on one condyle, a 2 mm shim was interposed to compensate for cartilage loss. For posterior femoral resections, posterior referencing was performed by contacting the most posterior points of the femoral condyles, similarly using a 2 mm shim when full-thickness posterior cartilage wear was present. In case of partial cartilage loss, remaining cartilage was removed with a curette.

Tibial resections were performed with manual instrumentation. Posterior slope was aligned parallel to the native slope of the medial tibial plateau, and coronal orientation (varus–valgus) was set to match the native proximal tibial joint line orientation. Coronal orientation and resection height were established using a measured-resection dual-stylus technique, with the objective of achieving a 10 mm tibial resection (minimum implant thickness). In the worn compartment, cartilage loss was assumed to be 2 mm and accounted for accordingly. Styluses were positioned centrally on each tibial plateau to mirror femoral landmark referencing and ensure symmetric resurfacing. When substantial tibial bone loss was suspected, bone loss was estimated clinically (eye-ball ± filler) after femoral trialing and osteophyte removal; tibial resection targets were adjusted accordingly.

### 2.3. Intraoperative Verification and Postoperative Digital Measurements

Intraoperatively, resection thickness was systematically verified using an analog caliper; when discrepancies from the planned thickness were deemed clinically relevant, corrective measures (e.g., recut for underresection, guide repositioning for overresection) were performed.

Immediately after surgery, the target thickness of each femoral and tibial resection was recorded by the senior surgeon in a standardized electronic data capture form. Target thickness was defined as implant thickness corrected for saw blade kerf (1.2 mm), cartilage loss (worn compartment only) and subjective estimation of bone loss (worn tibial compartment only). A cartilage thickness of 2 mm was assumed to represent full-thickness cartilage. Resection thicknesses were then measured by the senior consultant surgeon and a fellow using a digital vernier caliper (resolution 0.2 mm) at predefined locations, which were marked on the tibial cut surface using electrocautery: distal medial femur (DMF), distal lateral femur (DLF), posterior medial femur (PMF), posterior lateral femur (PLF), medial tibial plateau (MTP), and lateral tibial plateau (LTP). For tibial measurements on concave surfaces, peripheral elevated bone was carefully removed to allow proper seating of the caliper jaws. The digital caliper was systematically zeroed before each measurement series. Minimal systematic bias and high repeatability and reproducibility, with intraclass correlation coefficients greater than 0.95, have previously been reported for the femoral resection measurement technique used in this study.

The intra- and interobserver reliabilities of caliper measurements for medial and lateral tibial resections were assessed on 20 tibial bone specimens and found to be excellent. Intraclass correlation coefficients were 0.995 and 0.998 for intraobserver reliability and 0.986 and 0.987 for interobserver reliability, respectively. Excellent intra- and interobserver reliabilities of caliper measurements for femoral cuts were previously reported [8,9].

### 2.4. Statistical Analysis

All analyses were performed using postoperative digital caliper measurements of initial bone cuts performed with manual instrumentation, excluding any subsequent corrective cuts. Continuous variables are presented as mean ± SD (range) and categorical variables as *n* (%). Resection error was defined as measured minus target thickness (mm). Accuracy was expressed as mean signed error; precision was expressed as the SD of the signed error. Absolute errors were calculated. Errors were also categorized into predefined classes (<−2 mm, −2 to −1.5 mm, −1.5 to −1 mm, −1 to −0.5 mm, −0.5 to 0.5 mm, 0.5 to 1 mm, 1 to 1.5 mm, 1.5 to 2 mm, and > 2 mm). Mean signed errors and 95% confidence intervals (t-distribution) were displayed in forest-plot format. Agreement was assessed using Bland–Altman analysis (mean bias and 95% limits of agreement). Because of the retrospective design, no formal sample size calculation was performed. Statistical analyses and visualizations were performed using *Python* (version 3.11; Python Software Foundation, Wilmington, DE, USA) with *pandas*, *NumPy*, *SciPy*, and *Matplotlib* (*version 3.7.5*). *ChatGPT* (version GPT-5.3; OpenAI, San Francisco, CA, USA) was used for superficial text editing and generation of graphical elements.

## 3. Results

**Figure 1:**
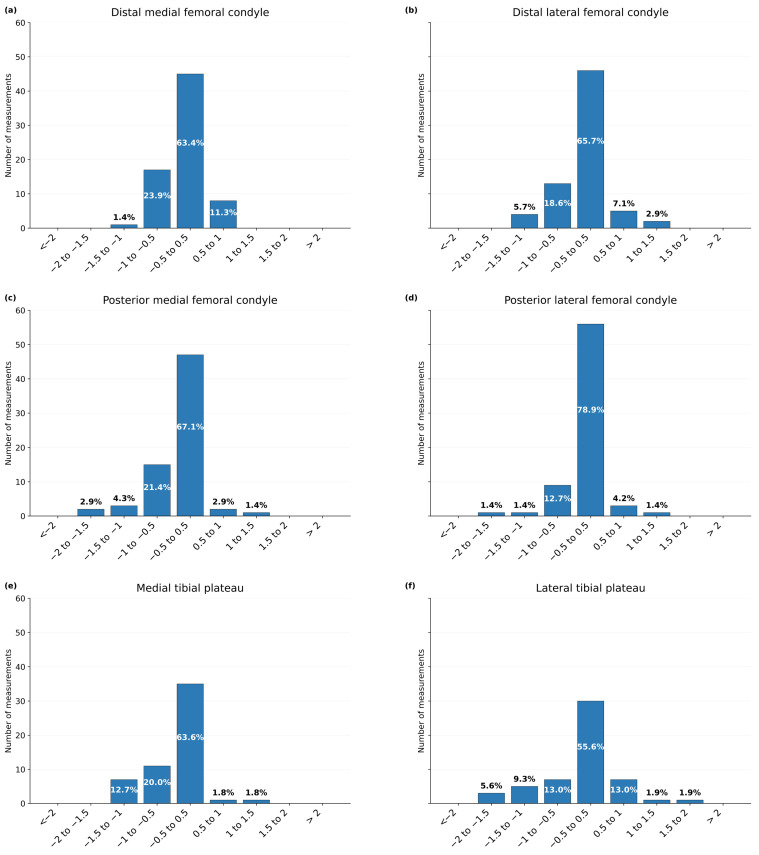
Distribution of resection error by predefined thickness classes. Error was defined as measured minus target resection thickness (mm). Bars represent the number of measurements within each predefined error class; percentages are displayed above each bar. Panels display (**a**) distal medial femoral condyle, (**b**) distal lateral femoral condyle, (**c**) posterior medial femoral condyle, (**d**) posterior lateral femoral condyle, (**e**) medial tibial plateau, and (**f**) lateral tibial plateau. Positive values indicate overresection and negative values underresection.

**Figure 2:**
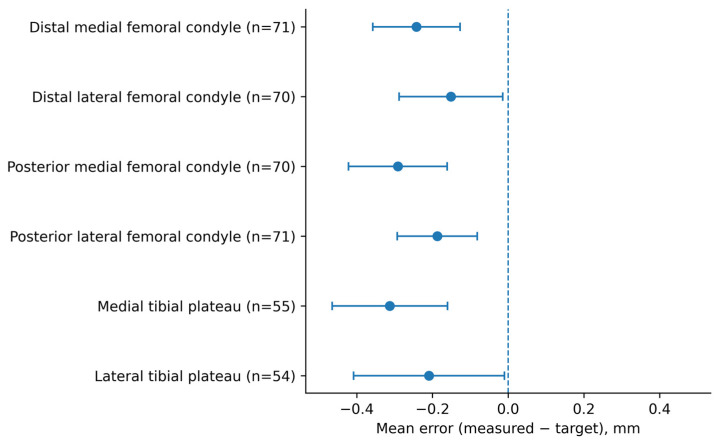
Mean signed resection error with 95% confidence intervals by resection site. Error was defined as measured minus target resection thickness (mm). Points represent mean signed error and horizontal lines represent 95% confidence intervals calculated using the t-distribution.

**Figure 3:**
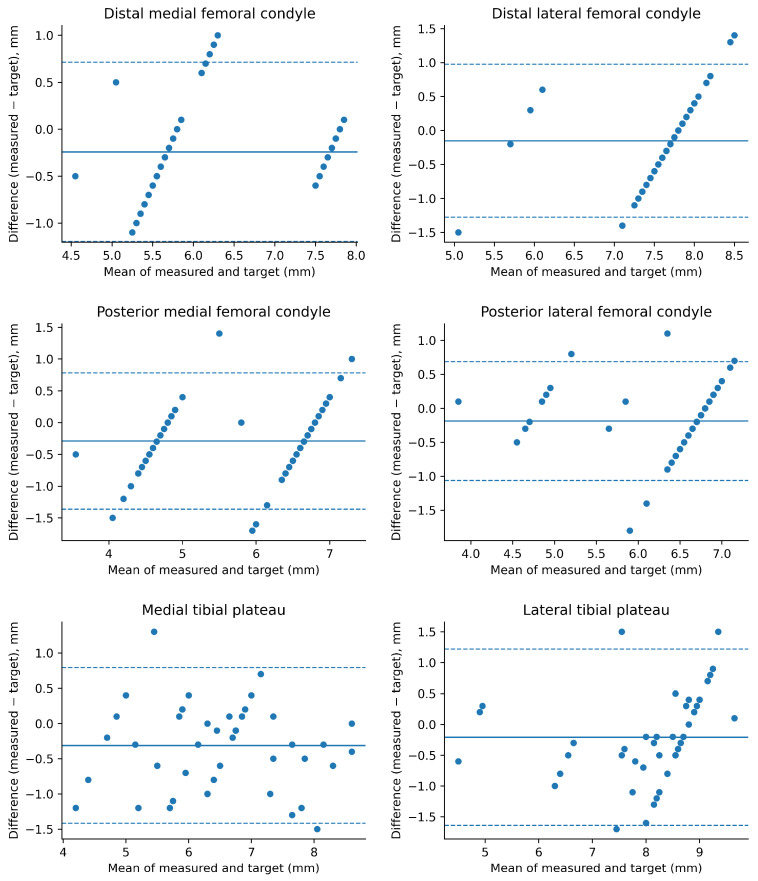
Bland–Altman analysis of resection thickness. Difference was defined as measured minus target resection thickness (mm). The solid horizontal line represents the mean bias and the dashed lines represent the 95% limits of agreement (mean ± 1.96 SD).

Femoral resections were assessed in 73 TKAs (292 femoral measurements) and tibial resections in 58 TKAs (116 tibial measurements), yielding a total of 408 measurements. Seventeen targeted thickness values were missing (two DMF, three DLF, three PMF, two PLF, three MTP, and four LTP), resulting in 391 resection sites included in the accuracy and precision analysis. Results are presented in Table 2 and Figure 1, Figure 2 and Figure 3.

**Accuracy and precision by resection site.** The mean signed error (measured − target) across all resections was low and consistently negative (−0.15 to −0.31 mm), indicating a small systematic underresection relative to target thickness. Precision, defined as the SD of the signed error, remained infra-millimetric across all sites (SD 0.45 to 0.73 mm). The lateral tibial plateau (LTP) exhibited the highest variability (SD 0.73 mm), whereas the remaining resections showed lower dispersion (SD approximately 0.45 to 0.57 mm, depending on the site). Mean absolute errors also remained low across sites (0.35 to 0.62 mm) with limited dispersion (0.31 to 0.43) (Table 2).

**Systematic bias.** Mean signed error by resection site is displayed in the forest plot (Figure 2). All resections demonstrated a small negative mean bias, consistent with systematic underresection relative to target thickness; the magnitude varied slightly between sites. The 95% confidence intervals (Figure 2) remained entirely below zero for each resection site, consistent with a statistically significant systematic bias.

**Error class distribution.** Class distribution analysis (Table 2 and Figure 1) showed that a relevant proportion of resections fell outside ±0.5 mm, ranging from 21.1% for PLF to 44.4% for LTP. Deviations outside ±1.0 mm were uncommon for femoral cuts (1.4% to 8.6%) but more frequent for tibial resections (14.5% for MTP and 18.5% for LTP). Importantly, no errors were observed outside ±2.0 mm.

**Agreement analysis.** Bland–Altman analysis (Figure 3) demonstrated a consistent negative mean bias across resection sites. Limits of agreement indicated that most measurements were distributed symmetrically around the mean bias, without evidence of marked heteroscedasticity.

## 4. Discussion

The most important finding of this study is that unrestricted KA TKA performed with manual instrumentation achieved high accuracy and infra-millimetric precision for both femoral and tibial resections when assessed using postoperative digital caliper measurements. Across 408 measurements, the mean signed error was consistently negative (−0.15 to −0.31 mm), indicating a small systematic underresection relative to target thickness, while precision remained infra-millimetric (SD 0.45 to 0.73 mm) across all sites. Mean absolute errors were low (0.35 to 0.62 mm), and no errors exceeded ±2.0 mm.

### 4.1. Femoral Resections

Femoral resections demonstrated low errors and limited dispersion, with mean absolute errors ranging from 0.35 mm (PLF) to 0.47 mm (PMF). The proportion of femoral resections outside ±1.0 mm remained limited (1.4% to 8.6%, depending on the site), supporting high reproducibility of the manual caliper-verified technique for initial femoral cuts.

The observed systematic underresection (negative mean signed error) may reflect several non-exclusive technical factors: (1) minimal bending of the 1.2 mm saw blade against hard subchondral bone; (2) built-in tolerance of the cutting guide’s saw slot, which is intentionally slightly wider than the blade to prevent jamming; and (3) the deliberate avoidance of compressing the distal femoral jig onto the distal femur by screw fixation. Collectively, these factors provide plausible explanations for the small but consistent negative bias, while the low SD values indicate that this bias was reproducible.

The femoral data presented in this study are comparable with those from previous investigations of the accuracy and precision of the analogic caliper-verified KA technique, which consistently demonstrated sub-millimetric accuracy of initial femoral resections [8,9,10,11]. Together these datasets demonstrate that the manual caliper-verified KA technique can reliably restore the native femoral joint line with high accuracy and reproducibility, without requiring frequent adjustments such as recutting in the case of significant undercut or shifting of the cutting guide posteriorly or distally in case of significant posterior or distal overcut, respectively.

### 4.2. Tibial Resections

Tibial resections also showed low absolute errors, with mean absolute error of 0.49 (0.42) mm for the medial tibial plateau and 0.62 (0.43) mm for the lateral tibial plateau. However, tibial resections exhibited a higher proportion of deviations outside ±1.0 mm (14.5% medial and 18.5% lateral) compared with femoral resections, suggesting a greater sensitivity of tibial resection thickness to technical variability.

Notably, the lateral tibial plateau resection was the least reliable, showing the highest variability (SD 0.73 mm) and the highest proportion outside ±0.5 mm (44.4%) and ±1.0 mm (18.5%). This finding is biomechanically plausible: the lateral plateau is further from the typically medially positioned cutting guide, therefore small micro-motion during sawing is expected to have a larger impact laterally than medially. This explanation aligns with the observed asymmetry in variability between MTP and LTP.

### 4.3. Clinical Relevance and Intraoperative Control

From a clinical standpoint, initial cut deviations do not necessarily translate into final implant malposition because resections are systematically verified intraoperatively with an analog caliper, and corrective actions can be performed when clinically relevant. This is particularly important for tibial resections, where thicker inserts, recuts, or other balancing strategies may compensate for important deviations. The present analysis quantifies the performance of initial bone cuts as assessed postoperatively with a high-resolution tool and should therefore be interpreted as a technical metrology benchmark rather than a direct surrogate for final component position.

### 4.4. Benchmark Versus Technology-Assisted TKA

The present results provide an important quantitative benchmark for evaluating technology-assisted TKA. Robotic-assisted and patient-specific instrumentation (PSI) systems are frequently justified by their purported ability to improve surgical accuracy. Nevertheless, the published literature on robot-assisted [12,13] and PSI-TKA [14,15] demonstrates greater variability across femoral resections (Table 3). This increased variability likely reflects cumulative error propagation related to registration, imaging acquisition, segmentation, guide positioning, and simplified cartilage thickness assumptions inherent to CT-based assistive technologies, which often assume uniform cartilage thickness across the joint.

The high accuracy and precision observed with the manual caliper-verified KA technique can be explained by the fact that KA is a measured-resection technique in which the expected thickness of each bone resection can be anticipated, without assuming cartilage thickness in unworn compartments. Indeed, the KA technique relies on simple, direct intraoperative bony referencing using readily accessible and reliable knee anatomic landmarks.

Comparisons with robotic-assisted and PSI studies should be interpreted cautiously, as these represent indirect cross-study comparisons with potential differences in measurement protocols. Therefore, the present findings should be considered as a benchmark rather than evidence of superiority over technology-assisted techniques.

## 5. Limitations

Several limitations must be acknowledged. Firstly, this study evaluated only the accuracy and precision of initial bone resections using manual caliper-verified KA and therefore does not inform final implant positioning, soft-tissue balance, or clinical outcomes. In practice, intraoperative adjustments (e.g., a 2 mm tibial recut following an equivalent underresection) allow close restoration of the native joint line height and orientation. Secondly, clinical outcomes were not evaluated, as this work was intentionally designed as a technical metrology analysis focusing on resection accuracy and precision, in line with comparable published studies that similarly do not report clinical outcomes. Thirdly, the retrospective design introduces potential selection bias. Indeed, some tibial resection thickness measurements may have been missed during the data collection period for various practical reasons (e.g., time constraints or inadvertent omission). Fourthly, as no formal comparison of baseline characteristics was performed between manually treated cases and those in which PSI was not used (mainly due to logistical constraints), a residual risk of selection bias cannot be excluded. However, given that allocation was not based on anatomical complexity and that the accuracy and precision of caliper-verified KA bone resections are primarily technique-dependent, baseline patient characteristics are unlikely to have influenced the tibial resection measurements. Fifthly, although each tibial and femoral resection is performed independently from a technical standpoint, multiple measurements were obtained within the same knee, introducing potential intraknee correlation. As such, observations may not be fully statistically independent, which could lead to an underestimation of variance. This should be considered when interpreting the precision estimates. Sixthly, postoperative measurements were performed by non-independent assessors without repeated measurements in the study dataset, which may introduce measurement bias despite previously demonstrated high reliability of the technique. Finally, the results were obtained by surgeons with extensive experience in the manual caliper-verified KA technique and using dedicated KA-optimized instrumentation. Surgeons early in their learning curve and/or using conventional (non-KA-optimized) manual instrumentation may achieve lower resection accuracy and precision, particularly for tibial resections, whereas the learning curve for femoral cuts in unrestricted KA has been shown to be short [10,11].

## 6. Conclusions

Manual caliper-verified unrestricted KA TKA achieved high accuracy and precision for both femoral and tibial resections within the described workflow, as assessed using postoperative digital caliper measurements. However, these findings do not establish superiority over other techniques and do not account for final implant position, soft-tissue balance, or clinical outcomes. This study provides quantitative data on tibial resection accuracy in uKA TKA and may serve as a benchmark for evaluating the performance of technology-assisted techniques.

## Figures and Tables

**Table 1:** Demographic, anthropometric, and anatomical characteristics of patients included in the digital measurement cohort. Preoperative alignment was assessed using the arithmetic hip–knee–ankle angle (aHKA), calculated as medial proximal tibial angle (MPTA) minus lateral distal femoral angle (LDFA). Constitutional coronal plane alignment of the knee (CPAK) classification was determined according to the method described by Bellemans et al., based on the combination of aHKA and joint line obliquity (JLO = MPTA + LDFA; apex distal < 177°, neutral 180° ± 3°, apex proximal > 183°). BMI, body mass index.

Sex (Male/Female)	35 (47.9%)/38 (52.1%)
Surgery Side (Left/Right)	37 (50.7%)/36 (49.3%)
Age (years)	Mean 71.15 (SD 7.92; 55 to 87)
BMI (kg/m^2^)	Mean 28.97 (SD 4.54; 19.57 to 43.34)
aHKA (°)	Mean −3.05 (SD 4.44; −11 to 9)
CPAK 1	34 (46.6%)
CPAK 2	16 (21.9%)
CPAK 3	5 (6.8%)
CPAK 4	10 (13.7%)
CPAK 5	4 (5.5%)
CPAK 6	3 (4.1%)
CPAK 7	1 (1.4%)
Posterior tibial slope (°)	Mean 7.64 (SD 3.64; 0 to 17)

**Table 2:** Error was defined as the difference between measured and target resection thickness (measured − target, mm). Accuracy was expressed as the mean signed error and precision as the standard deviation (SD) of the signed error. Absolute error values were also calculated. For each predefined threshold (±0.5 mm, ±1.0 mm, ±1.5 mm, and ±2.0 mm), the proportion of resections outside the threshold is reported, together with the directional distribution. Values are presented as number of measurements, mean, standard deviation, minimum and maximum, or percentage (number) as appropriate. DMF: Distal medial femoral; DLF: Distal lateral femoral; PMF: Posterior medial femoral; PLF: Posterior lateral femoral; MTP: Medial tibial plateau; LTP: Lateral tibial plateau.

	DMF	DLF	PMF	PLF	MTP	LTP
**Absolute error (mm): mean (SD)**	0.44 (0.31)	0.45 (0.38)	0.47 (0.41)	0.35 (0.33)	0.49 (0.42)	0.62 (0.43)
**Outside ±0.5 mm**	36.6% (26)	34.3% (24)	32.9% (23)	21.1% (15)	36.4% (20)	44.4% (24)
<−0.5 mm	25.4% (18)	24.3% (17)	28.6% (20)	15.5% (11)	32.7% (18)	27.8% (15)
>+0.5 mm	11.3% (8)	10.0% (7)	4.3% (3)	5.6% (4)	3.6% (2)	16.7% (9)
**Outside ±1.0 mm**	1.4% (1)	8.6% (6)	8.6% (6)	4.2% (3)	14.5% (8)	18.5% (10)
<−1.0 mm	1.4% (1)	5.7% (4)	7.1% (5)	2.8% (2)	12.7% (7)	14.8% (8)
>+1.0 mm	-	2.9% (2)	1.4% (1)	1.4% (1)	1.8% (1)	3.7% (2)
**Outside ±1.5 mm**	-	-	2.9% (2)	1.4% (1)	-	7.4% (4)
<−1.5 mm	-	-	2.9% (2)	1.4% (1)	-	5.6% (3)
>+1.5 mm	-	-	-	-	-	1.9% (1)
**Outside ±2.0 mm**	-	-	-	-	-	-

**Table 3:** This table compares the accuracy and precision of femoral bone resections achieved with manual caliper-verified kinematic alignment total knee arthroplasty (present study), robot-assisted TKA, and patient-specific instrumentation (PSI) TKA. Mean absolute error ± standard deviation, expressed in millimeters. DMF: Distal medial femoral; PMF: Posterior medial femoral; DLF: Distal lateral femoral; PLF: Posterior lateral femoral.

Technique/In Vivo Study	N	DMF	PMF	DLF	PLF
Present study	73	0.44 ± 0.31	0.47 ± 0.41	0.45 ± 0.38	0.35 ± 0.33
**Robot-assisted TKA**					
Rossi et al. [13]	75	0.8 ± 0.6	0.4 ± 0.6	0.9 ± 0.7	0.6 ± 0.5
Gamie et al. [12]	44	0.7 ± 0.6	1.1 ± 1.0	0.9 ± 1.2	1.0 ± 0.8
**PSI-TKA**					
Wernecke et al. [15]	118	0.9 ± 1.3	1.5 ± 2.1	0.9 ± 1.3	0.8 ± 1.2
Yamamura et al. [14]	45	0.6 ± 0.7	1.8 ± 0.9	1.4 ± 1.2	1.7 ± 1.0

## Data Availability

The data that support the findings of this study are available from the corresponding author, C.R., upon reasonable request.

## Abbreviations

KA	kinematic alignment
TKA	total knee arthroplasty
uKA TKA	unrestricted kinematic alignment total knee arthroplasty
DMF	distal medial femur
DLF	distal lateral femur
PMF	posterior medial femur
PLF	posterior lateral femur
MTP	medial tibial plateau
LTP	lateral tibial plateau
FA	functional alignment
PSI	patient-specific instrumentation
